# Novel stereo DIC characterisation of microneedle and hypodermic needle insertion

**DOI:** 10.3389/fbioe.2025.1580464

**Published:** 2025-06-30

**Authors:** Megan McNamee, Thomas Pritchard, Jacob Mitchell, Chris Bolton, Kerry Roberts, Owen Guy, Huma Ashraf, Hari Arora

**Affiliations:** ^1^ Biomedical Engineering Simulation and Testing Laboratory, Swansea University, Swansea, United Kingdom; ^2^ Materials and Manufacturing Academy, Swansea University, Swansea, United Kingdom; ^3^ KLA Corporation, Newport, United Kingdom; ^4^ Department of Chemistry, Swansea University, Swansea, United Kingdom

**Keywords:** microneedle (MN), DIC (digital image correlation), strain, skin phantom, needle insertion

## Abstract

**Introduction:**

Microneedles are minimally invasive devices, designed for pain-free drug delivery. Until now, the degree of strain exerted on the skin during microneedle insertion, in comparison to gold standard hypodermic needles, has not been quantified.

**Methods:**

This paper presents experimental results from a novel digital image correlation setup to quantify maximum normal strain exerted on a skin-mimicking membrane by hollow silicon microneedles and 25-gauge stainless steel hypodermic needles through contact, deformation, rupture, and device insertion.

**Results:**

Findings here have shown 1 × 5 hollow silicon microneedle arrays exert significantly lower maximum normal strain compared to 25-gauge hypodermic needles. There is an average of 75% decrease in the maximum normal strain experienced by the membrane when using microneedle devices in comparison to that of the 25-gauge hypodermic needles. This quantification of strain has been discretised to each individual needle in the microneedle device, allowing for informed design choices for future device iterations.

**Discussion:**

These findings suggest the hollow microneedle devices to be a gentler alternative for transdermal applications, potentially improving patient comfort and reducing tissue trauma when compared to the gold standard, traditional 25-gauge hypodermic needle.

## Introduction

### Microneedles

**FIGURE 1:**
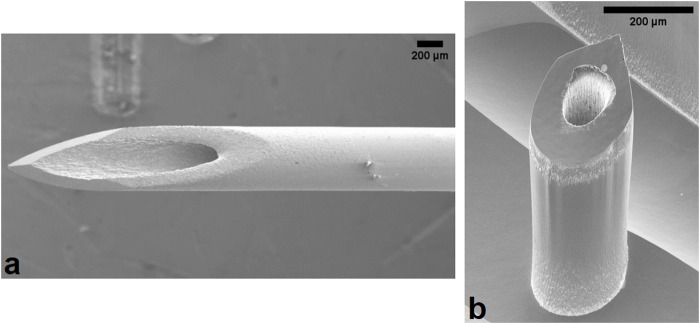
Scanning electron microscope image of the **(a)** 25G hypodermic needle and **(b)** hollow silicon microneedle used in this experiment. One microneedle device is a linear array comprised of 5 individual microneedles. Scale bar represents 200 μm.

Following their initial conception in 1976, microneedles (MN) are a classification of medical devices which are garnering huge interest across both the medical and public sectors through the widespread reporting of dose sparing potential and pain-free, minimally invasive injections. Their first microfabrication was published in 1998 by Henry et al., who successfully reported transdermal calcein delivery via coated solid silicon MNs. More than 2 decades later, the field has seen the marked development of MNs with respect to material, size, structure, and function. There is a variety of MN types now available including solid, dissolving, coated, swellable, and hollow. Hollow silicon microneedles (Figure 1b) used in this study were fabricated using easy to scale semiconductor fabrication techniques, based on that reported by Bolton et al. (2020). Further characteristics of MN devices can be altered to enable better biological targeting, such as variations in height allowing insertion depth to be altered to better target specific epidermal and dermal layers, enabling more precise delivery to Langerhans, macrophages, and dendritic cells, resulting in a more efficacious response.

MNs were conceptualised as pain-free, minimally invasive drug delivery and sensing devices, which elicit a reduced level of anxiety for patients, especially those within paediatric and trypanophobia-afflicted populations. In addition to these more qualitative and holistic benefits, MN devices have been reported to hold significant dose sparing potential. MN show potential advantages in dosing efficacy, stability, and sterility particularly within the immunotherapeutic and vaccination field (Quan et al., 2010; Van Damme et al., 2009; Menon et al., 2021).

### Pain measurement

The qualitative advantages of MN have been extensively reported, with the most widely referenced claim being that of “pain-free” drug delivery. Human studies and clinical trials have been performed employing the Visual Analogue Scale (VAS). In addition, the Sensation/Pain Rating Index (Haq et al., 2008; Gill et al., 2008) was used to assess pain intensity and quality, striving to enable a comprehensive assessment of patient experience. These methods, however, inherently integrate subjectivity into the assessment. Despite its usability and simplicity cementing the VAS as a valuable tool in many clinical and research settings, there are a considerable number of limitations, detailed in Table 1, which necessitate the use of supplementary methods to ensure reliable and quantitative measurement of pain related experiences.

**TABLE 1:** A summary of the flaws and limitations associated with the use of the visual analogue scale for pain quantification.

Flaw	Limitations	
Subjectivity and variability	*Interpretation variability*	Varying interpretations of scale endpoints
	*Personal differences*	Variation in pain tolerances and thresholds
Lack of precision	*Difficulty in marking*	Difficulties with identifying precise points on scale deceasing accuracy
	*Data resolution*	Difficulty in converting marks from a 1–10 cm line to a 1–100 numerical value
Cognitive and physical limitations	*Geriatric and paediatric populations*	Individuals within these populations may struggle with understanding consistent use of the scale
	*Cognitive impairment*	Individuals within these populations may struggle with accurate interpretation and use of the scale
	*Physical disabilities*	Individuals with motor impairments may find marking accurately challenging
Cultural and language differences	*Cultural perceptions*	Cultural differences in expressing pain and other subjective experiences can affect VAS use
	*Language barriers*	Variations in language comprehension can contribute to differences in scale interpretation
Emotional and psychological influences	*Emotional state*	Fluctuations in a personal psychological and emotion state can influence pain interpretation
	*Response bias*	Pain over/underestimation due to internal biases, e.g., stoicism
Anchoring effects	*Contextual*	Previous experiences and expectations may influence how individuals rate pain/experiences
Lack of standardisation	*Different versions*	Variations in wording and scale length can lead to inconsistencies across studies
Non-linearity	*Non-linear perception*	Relationships between marks on the line and perceived pain intensity is not always linear
Analysis and interpretation challenges	*Statistical treatment*	VAS data are continuous, making their interpretation and statistical treatment challenging

Whilst the VAS relies on subjective patient reporting, it leaves room for methodologies that more reliably and accurately quantify experiences associated with pain. Quantifying strain during needle insertion would offer an objective and measurable approach to pain assessment, enabling consistent, comparable, and reliable data collection. This has been supported by papers such as those published by Urits et al., who have highlighted the need for more objective diagnostic measures such as strain quantification for subjective pain scales. Furthermore, they highlighted the need for integrating strain measurement techniques into clinical practice to improve end user experiences (Urits et al., 2019).

### Biological strain and pain

As the body’s largest organ, and its primary interface with the environment, the skin experiences diverse strains from everyday activities and injuries. Biological strain experienced by the skin plays a pivotal role in the sensation of pain through a variety of mechanisms, intricately linking mechanical stressors to sensory responses, with the ability to sense touch facilitated by the somatosensory system. When the skin is subject to sensations such as pressure, temperature, and location-based stimuli, specialised receptors termed mechanoreceptors, thermoreceptors, nociceptors, and proprioceptors respectively respond accordingly. Upon tissue damage, the body’s natural response to injury involves the activation of pain receptors and the release of inflammatory mediators like histamines and cytokines, which locally increase blood flow and recruit immune cells, but also activate specific receptor subtypes to influence the resultant signalling cascade to the brain. For example, traditionally used hypodermic needles (Figure 1a) insert into the dermal layers of the skin, where Pacinian corpuscles, Ruffini corpuscles, and larger nerve endings reside. Conversely, MNs insert into the epidermis, interacting predominantly with Merkel’s disc, housed between the stratum basale and papillary dermis. These small, finely calibrated mechanoreceptors are the cells which predominantly are activated following MN insertion, due to their responsibility for the identification of sustained light pressure in hairy and non-hairy skin. While Merkel’s disc and Meissner corpuscles are sensitive to initial mechanical deformation, increasing levels of strain are required to activate nociceptive fibres (A-δ and C fibres), which are responsible for pain perception. Needle insertion into the epidermis induces strain, particularly during puncture and deeper insertion as facilitated by hypodermic needles, which could explain the transition from mechanoreception to nociception and the onset of pain. Excessive strain generated by more invasive medical devices can lead to micro-injuries and inflammation, heightening nociceptor sensitivity and intensifying pain responses. Liu et al. (2023), found higher strain rates correlate to greater tissue damage and they concluded that this led to greater pain using the VAS. Understanding this intricate relationship is crucial across medical and clinical settings.

Moreover, the connection between biological strain and pain perception underscores the complex interplay of mechanical forces, sensory pathways, and physiological responses in the human body. By advancing our understanding of pain mechanisms, researchers and healthcare professionals can refine interventions to alleviate pain, improve quality of life, and optimize health outcomes for individuals.

Medical devices, such as needles, cause biological strain on the skin and lead to pain through direct mechanical disruption and subsequent inflammatory responses. When a needle punctures the skin, it creates a small but significant breach in the epidermal and dermal layers. This puncture disrupts the integrity of skin tissues, causing immediate mechanical strain as well as the redistribution of naturally occurring strain. Studies by Arendt-Nielsen et al. (2006) have studied skin reaction and VAS to record pain intensity and unpleasantness during insulin injections with various hypodermic needles. This study reported the use of thinner hypodermic needles with a higher gauge number registered lower on the VAS pain intensity and unpleasantness scale, supporting the use of higher gauge needles for pain reduction (A-N et al., 2006). Moreover, repeated or prolonged use of needles, such as in intravenous therapies or insulin injections, can exacerbate tissue strain and inflammation leading to chronic discomfort and potential skin damage over time. This, in addition to studies by Shergold and Fleck (2004) who quantitatively studied the reliance of force required on punch geometry, supports further use of quantitative measures for strain, and resultant pain, following hypodermic needle and MN mediated injections.

### Digital image correlation

Digital Image Correlation (DIC) is an optical surface measurement technique renowned for its non-contact measurement capabilities in quantifying strain within materials. This methodological approach to strain assessment is pivotal, as it captures the nuanced deformations that occur when materials respond to applied stresses. DIC operates by tracking the displacement of surface patterns during deformation, thereby enabling comprehensive full-field strain measurements across a surface. However, achieving accurate results hinges significantly on precise control and optimisation of various parameters such as speckle patterns, lighting conditions, and camera settings, which influence the sensitivity and reliability of DIC measurements (Jones and Iadicola, 2018; Wang et al., 2024a; Lecompte et al., 2006).

DIC methodologies are diverse, accommodating different testing scenarios: 2D DIC, ideal for planar test specimens that observe no out-of-plane motion during the test, and stereo-DIC, which utilises multiple cameras to facilitate 3D coordinate measurements and displacement analysis. DIC plays a crucial role in material testing, where it provides insights into how materials respond to varying loads, their failure conditions, and performance benchmarks, including biomechanics applications. Moreover, DIC in addition to other imaging methods, such as shape analysis via scanning and 3D optical profilometry, are indispensable in production quality control settings, where it aids in defect detection and validation of manufacturing processes.

Despite its numerous strengths, DIC is not without its challenges. It is highly sensitive to environmental factors such as lighting and speckle quality. These conditions must be optimised for the application required, to ensure consistent and repeatable results.

DIC was used in this study to measure the deformation and puncture of skin phantoms following both hypodermic needle and MN mediated insertion, quantifying this deformation with respect to maximum normal strain. This study has been performed with a vision of quantifying, in an objective and repeatable manner, the strain experienced by a skin phantom during insertion of MNs in comparison to traditional hypodermic needles.

## Materials and methods

### Materials

#### Membrane

A silicone 0035 was mixed with a black silicone pigment to produce a completely opaque, epidermal-mimicking membrane. To ensure the membrane closely mimics the mechanical properties of human epidermis, the silicone was selected based on its elastic modulus of 0.125 MPa, which is comparable to the reported value of 0.1 MPa for epidermis, making it a suitable model for simulating skin-like behaviour in this study (Shintake et al., 2017; Kalra et al., 2016). The silicone was bar coated to achieve a consistent membrane thickness of 50 μm and left overnight to ensure a complete and homogenous cure. Samples were then airbrushed with a mixture of white pigment and acrylic paint thinner to produce white speckles on the black membrane.

#### PDMS preparation

An underlying structure was required to mimic the support structure present under the skin, naturally comprising of a mixture of bone, muscle, and fat. To mimic this, poly (dimethylsiloxane) (PDMS) was used (Huang et al., 2010). The use of PDMS for these purposes has been previously reported by Huang, Lee and Li (2010), and Balaban et al. (2001), describing the flexible elastomer as deformable and also able to simulate mechanically loaded muscle environments *in vivo*. Furthermore, imaging was performed on the underside of the membrane through the support structure, therefore it was critical for this to be optically clear to prevent abnormalities in speckle tracking during insertion. PDMS is highly regarded for its optical transparency, and therefore meets the criteria for the support system required (Miranda et al., 2021; Reidy et al., 2018). To produce the PDMS support network, 50 mL PDMS *per* mold was produced with 8% crosslinker to best yield the appropriate elasticity range (∼200–500 kPa) for the dermal support system mimic, and was poured into a silicone mold designed as a negative of the void area seen in the ring of the testing support setup (Wang et al., 2024b). An acrylic disc was placed at the bottom of the mold to ensure a flat base, ease of removal, and limit artifacts on the imaged surface.

### Apparatus

#### Membrane mount and mirror apparatus

**FIGURE 2:**
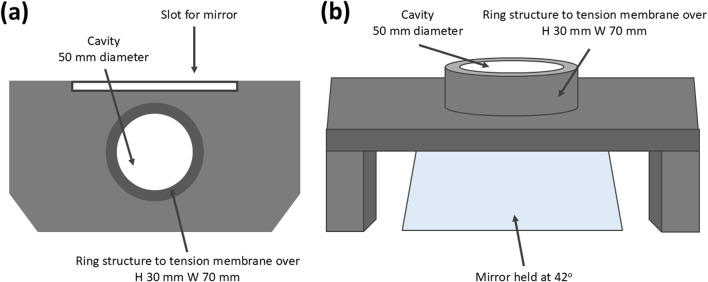
**(a, b)** Resin printed mounting apparatus to house the mirror and multiaxially tension the skin phantom (ring H 30 mm W 70 mm cavity W 50 mm).

A mounting apparatus was designed to replace the bottom platen of a force station, allowing the transparent PDMS plug structure to be placed into the cavity within the ring, and the membrane was laid flush over this, speckle side down, and tensioned over the ring prior to needle insertion (Figures 2a, b). The mounting apparatus was designed with a 42^o^ slot for a mirror to enable imaging from the underside to capture penetration point. The mount was 3D printed in clear resin using a FormLabs 3B 3D printer.

#### Mountings

**FIGURE 3:**
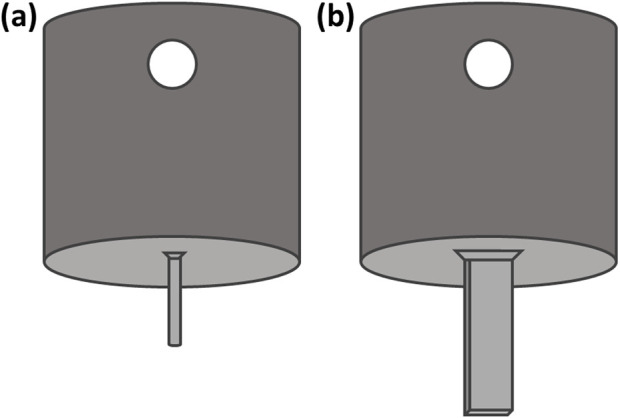
**(a)** Hypodermic needle mount to allow mounting onto the mechanical testing machine via a push Luer attachment **(b)** Microneedle mount with flat surface at the end for sample mounting (L 7.5 mm, W 1.60 mm). Dimensions of the mounting surface are equal to that of the microneedle array base.

Mounts for needle samples were designed to fit samples to the mechanical testing machine used (Tinius Olsen mechanical testing machine Model 1ST) and to ensure limited interference between the mounting system and the membrane during insertion (Figure 3a, b).

### Testing methods

#### Mechanical testing method

A Tinius Olsen Model 1ST mechanical testing machine was used to drive samples into the membrane in a displacement controlled manner and collect quantitative force-displacement data. A 25N load cell was used, with a 0.001 N initiation value, whereby contact was reliably detected during trials. The noise floor of the load cell fell below the test initiation value. A 20 N load protection limit was applied. The insertion speed of the needles was controlled by the head speed movement, fixed at 1 mm/s.

#### Camera setup and DIC method

**FIGURE 4:**
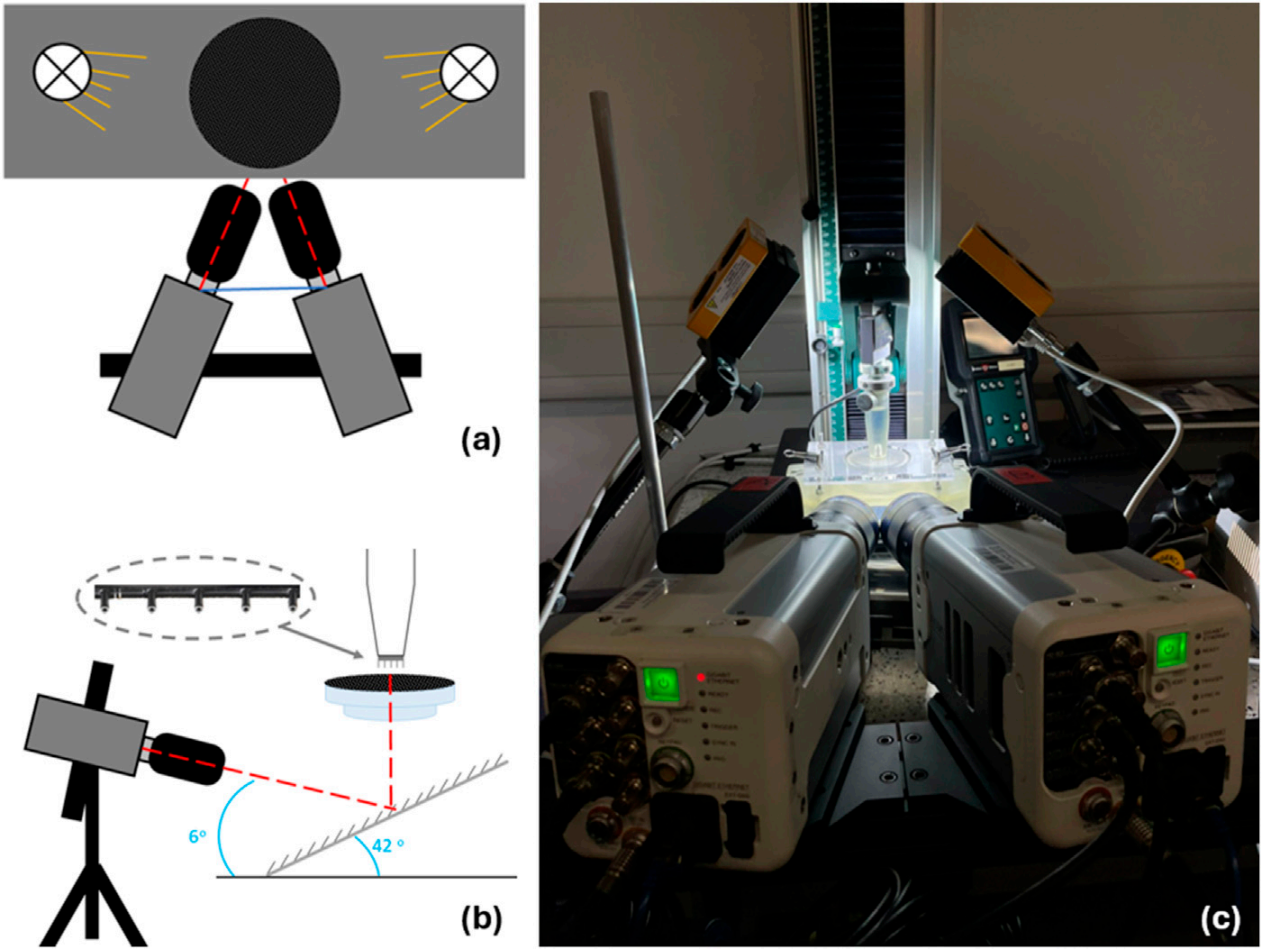
Insertion and imaging setup. **(A)** top down and **(B)** side on schematic visualising needles inserted perpendicular to skin mimic to replicate intramuscular injections. Insert can be seen to highlight location of microneedles on mounting apparatus. **(C)** image of insertion experiment including cameras, lights, and support bench with skin phantom clamp.

The camera and lighting required for the study comprised of two Photron FASTCAM Nova S6 high-speed cameras with 100 mm lenses and linear polarising filters, and two GS Vitec MultiLED QT light sources (Figure 4; Table 2). The light sources were fixed above the mounting apparatus, angled down at the membrane (Figure 4). The two high-speed cameras were fixed at an angle of incline of 6^o^ at the mirror. All parameters applied during DIC processing can be found in Table 3.

**TABLE 2:** Camera and digital image correlation measurement parameters and values applied for the experimental setup.

	Parameter	Value
Camera	*Manufacturer model*	Photron FASTCAM Nova S6
	*Lens make and model*	Laowa 100 mm f/2.8 2x Ultra Macro APO
	*Frame rate*	1,000 fps
	*Shutter speed*	1/3,000 s
	*Resolution*	1,024 × 1,024
	*Total frames*	5,437
	*Trigger mode*	Manual, 10% pretrigger
	*Aperture*	8
DIC Measurement	*Focal length*	100 mm
	*Incline angle*	6^o^
	*Stereo angle*	33^o^
	*SOD*	310 mm
	*DOF*	3 mm
	*FOV*	30 mm
	*Mirror angle*	42^o^

**TABLE 3:** Digital image correlation processing parameters applied during image processing.

DIC processing parameter	Value
*Software package name and manufacturer*	LaVision DaVis 10 StrainMaster
*Subset size*	19
*Step size*	12
*Minimum number of value pixels*	100%
*Pyramid levels*	1
*Maximum iterations*	30
*Epsilon*	30
*Threshold for correlation value*	0.5
*Threshold for confidence margin*	0.25 pixels
*Threshold for triangulation error*	3 pixels
*Interpolation mode*	Bilinear
*Noise floor*	2.08E-13%

## Results

### Force–displacement

**FIGURE 5:**
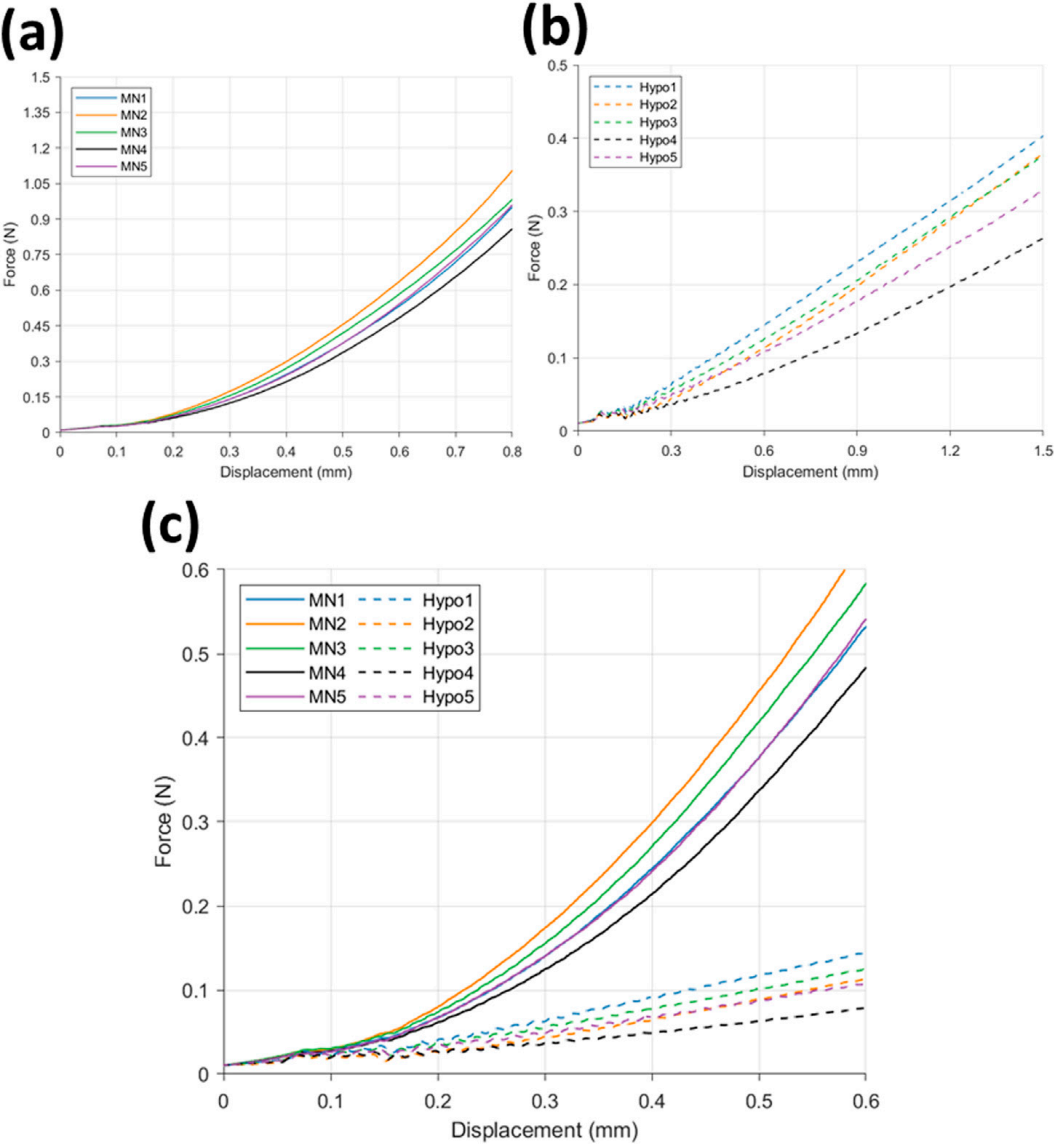
Force displacement curve for **(a)** microneedle (*n* = 5) and **(b)** hypodermic needle (*n* = 5) insertions, and **(c)** a comparison of microneedle (−) (n = 5) and hypodermic needle (--) insertions (n = 5).

Force-displacement data for MN insertion into the membrane/PDMS support system can be seen in Figure 5a. The trend depicts a gradual increase in force between 0–0.15 mm displacement, reaching a maximum force of 0.049 N, followed by a greater increase to 1.1 N at 0.8 mm displacement. This trend is consistent between the 5 repeats, with samples MN2 and MN4 registering the maximum and minimum measured force values respectively.

The hypodermic needle insertion force-displacement graph (Figure 5b) depicts a greater variation in force during insertion between 0–0.15 mm. More specifically, an initial climb in force up to 0.033 N can be observed between 0–0.14 mm, followed by a decline to 0.025 N at 0.15 mm. Although a small decrease in force, this drop off is seen consistently across all 5 repeats, showing good repeatability and indicating this to be related to the insertion and membrane rupture itself, as opposed to noise. Rupture denotes the moment the needle tip breaks through the skin-mimicking membrane, marking the point of insertion and surface failure.

A comparison of the force-displacement graphs for MN (−) and hypodermic needle (--) insertion (*n* = 5 per device type) can be found in Figure 5c. The comparison allows the identification of the clear differences in force required for insertion, specifically depicting the MN devices recording a greater force during insertion than the hypodermic needle devices. Similar forces are recorded between 0–0.15 mm for both device types, before a distinctive split is seen with MN induced force increasing with a faster rate than the more gradual increase in force observed in the hypodermic needle sample results.

### Digital image correlation

**FIGURE 6:**
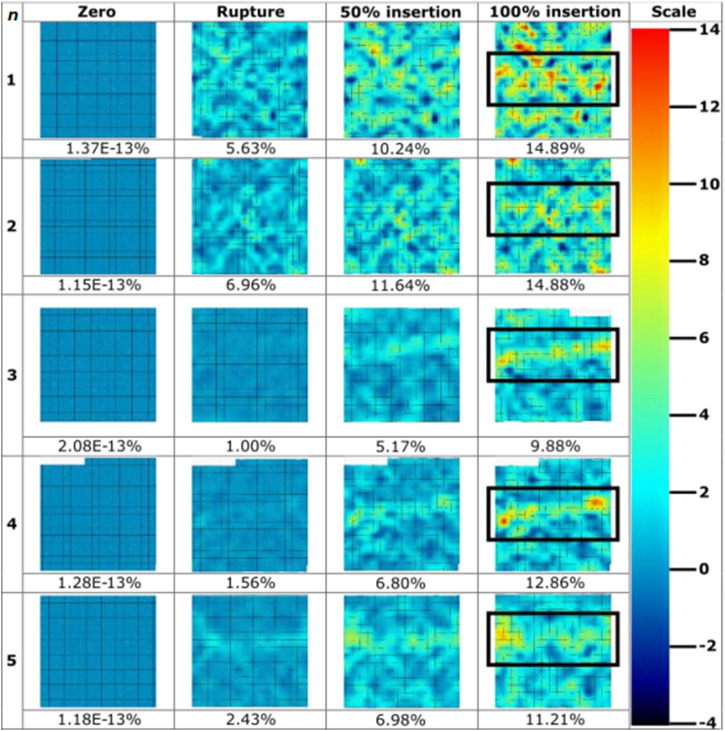
Strain field images throughout microneedle insertions (*n* = 5) extracted throughout the course of insertions. Values for maximum normal strain experienced at imaged frames are pictured below the strain field, with colour bar scale provided. Microneedle array at 100% insertion frame has been boxed for ease of identification.

Strain fields were calculated as per the parameters detailed in Table 4. MN-induced strain field images were extracted at four significant frames during the tests: zero; membrane rupture; 50% insertion depth; and at 100% insertion depth (Figure 6). The % depth refers to the insertion depth of the microneedle, expressed as a percentage of the total needle length. A scale of -4 – 14% maximum normal strain was used, notably seeing slight saturation within the visualisation of the maximum strain value for the sample sets (MN1, 14.89%; MN2, 14.88%) (Figure 6). This was allowed as it enabled the best visualisation the entirety of the dataset. The average maximum normal strain and standard deviation for each of these 4 frames are 1.41E-13% (3.86E-14%), 3.5% (2.6%), 8.2% (2.7%), and 12.7% (2.2%) (Figure 6). The maximum strain induced at rupture by MN devices is 27.6% of that induced at full insertion. Visually, as the test progresses, hot spots in a linear orientation can be identified across each sample where the MN 1 × 5 array inserted (Figure 6). This has been located with a black rectangle for ease of identification. The maximum normal strain ranged from 1.00% to 6.69% at the point of MN-facilitated rupture, with an average of 3.52% (Figure 6). At 100% MN insertion, the maximum normal strain range is 9.88%–14.89%, with the average being 12.74% (Figure 6) (Table 4).

**TABLE 4:** Tabular summary of the minimum, maximum, and average maximum normal strain exerted by each device type at the four points of data extraction.

	Maximum normal strain (%)							
	*Microneedles*				*Hypodermic Needle*			
	*Zero*	*Rupture*	*50%*	*100%*	*Zero*	*Rupture*	*15%*	*30%*
Min	1.15E-13	1.00	5.17	9.88	1.04E-13	0.64	14.77	34.13
Max	2.08E-13	6.96	11.64	14.89	2.89E-13	2.20	30.99	67.08
Mean	1.41E-13	3.52	8.17	12.74	1.61E-13	1.59	23.22	51.43

**FIGURE 7:**
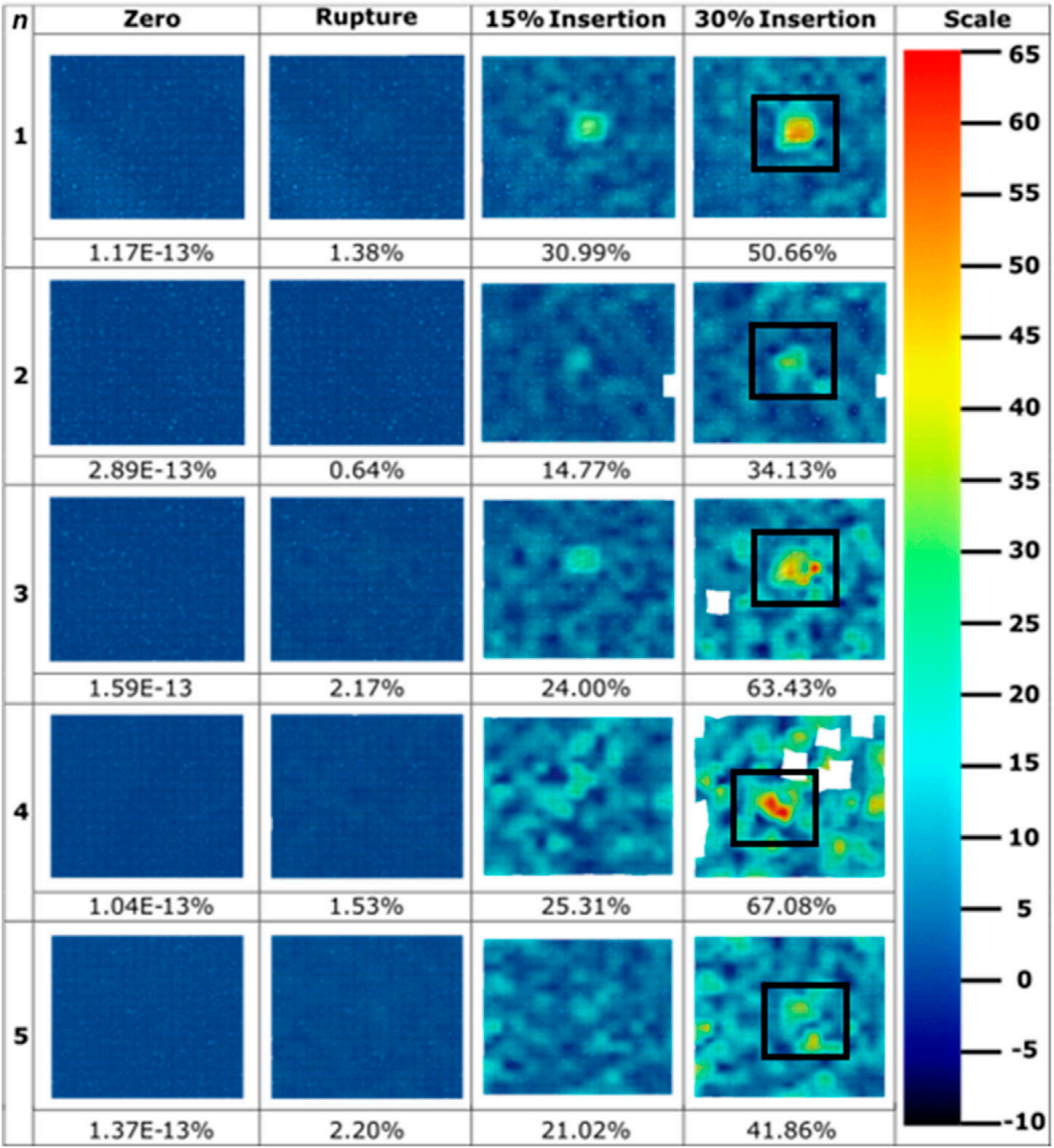
Strain field images throughout hypodermic needle insertions (*n* = 5) extracted throughout the course of insertions. Values for maximum normal strain experienced at imaged frames are pictured below the strain field, with colour bar scale provided. Sample 3’s final image was extracted at 29% insertion due to facet loss. Hypodermic needle at 30% insertion frame has been boxed for ease of identification.

Hypodermic needles induced strain field images were extracted at four significant frames during the tests: zero; membrane rupture; 15% insertion depth; and at 30% insertion depth (Figure 7). A scale of -10%–65% maximum normal strain was used, notably seeing saturation at the maximum strain value for the samples set (hypodermic needle repeat 5, 72.7%) to best visualise the entirety of the dataset. The average maximum normal strain and standard deviation for each of these 4 frames are 1.61E-13% (7.44E-14), 1.59% (0.64), 23.22% (5.95), and 51.43% (13.97) (Figure 7). Notably, the average maximum normal strain for rupture is significantly smaller at 1.59%, just 3.09% of that induced at 30% insertion depth. Visually, the high strain can be seen in each sample to be highly localised to the tip area of the needle with limited effect on the surrounding membrane. It is important to note that sample 3’s final image was extracted at 29%, not 30%, insertion due to facet loss at this frame number which may have been contributed to by factors such as shadows, object interference in the optical path, or excessive deformation, which hindered the DIC algorithm’s ability to track the facet.

**FIGURE 8:**
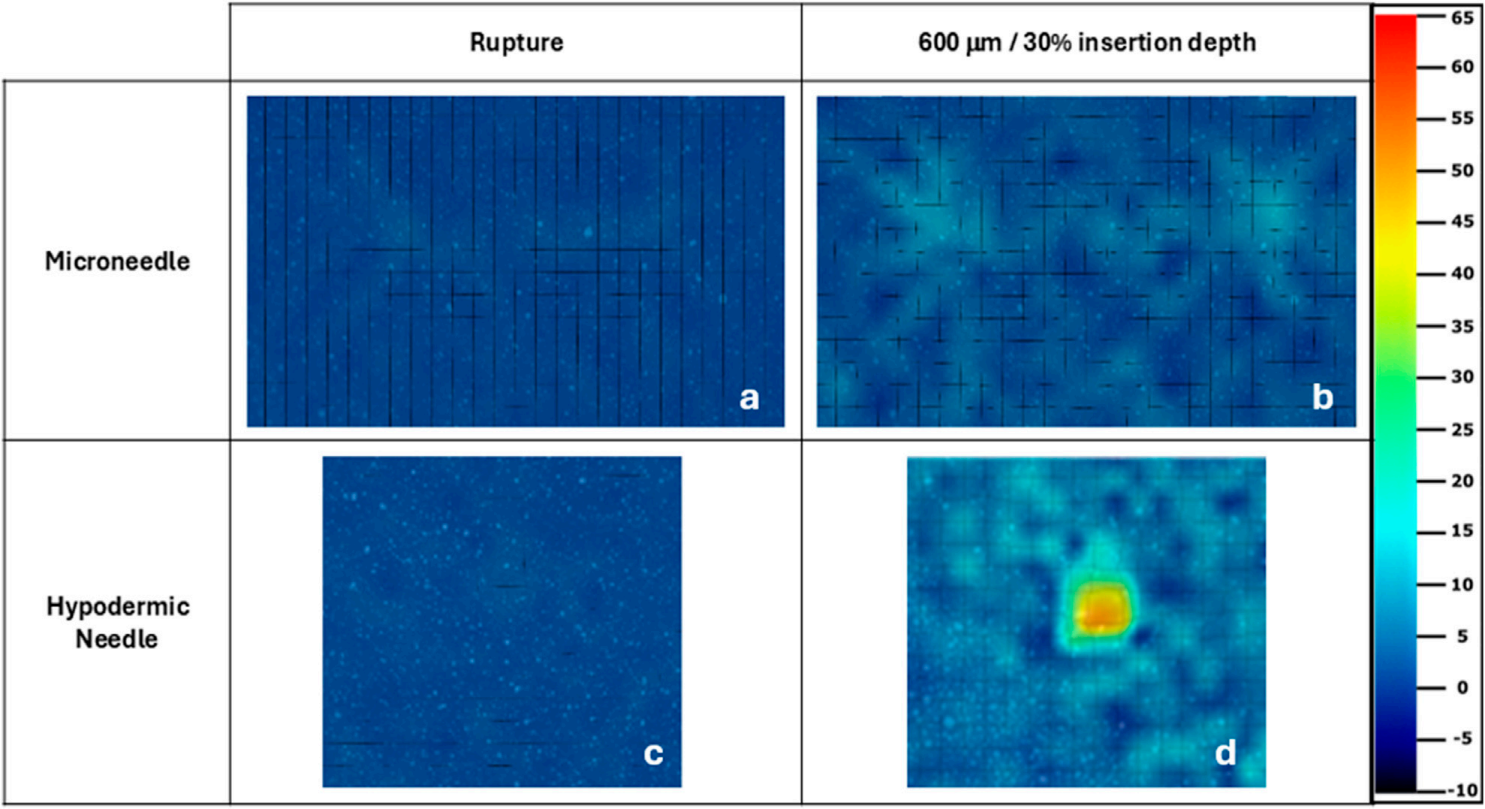
Microneedle **(a, b)** and hypodermic needle **(c, d)** strain fields for maximum normal strain experienced at rupture and full/30% insertion depth respectively. The same strain colour scale used for the hypodermic needle samples in Figure 6 has been applied to all samples strain fields. The microneedle and hypodermic needle sample selected here were both the samples with the median result to ensure good representation of the complete sample sets.

**FIGURE 9:**
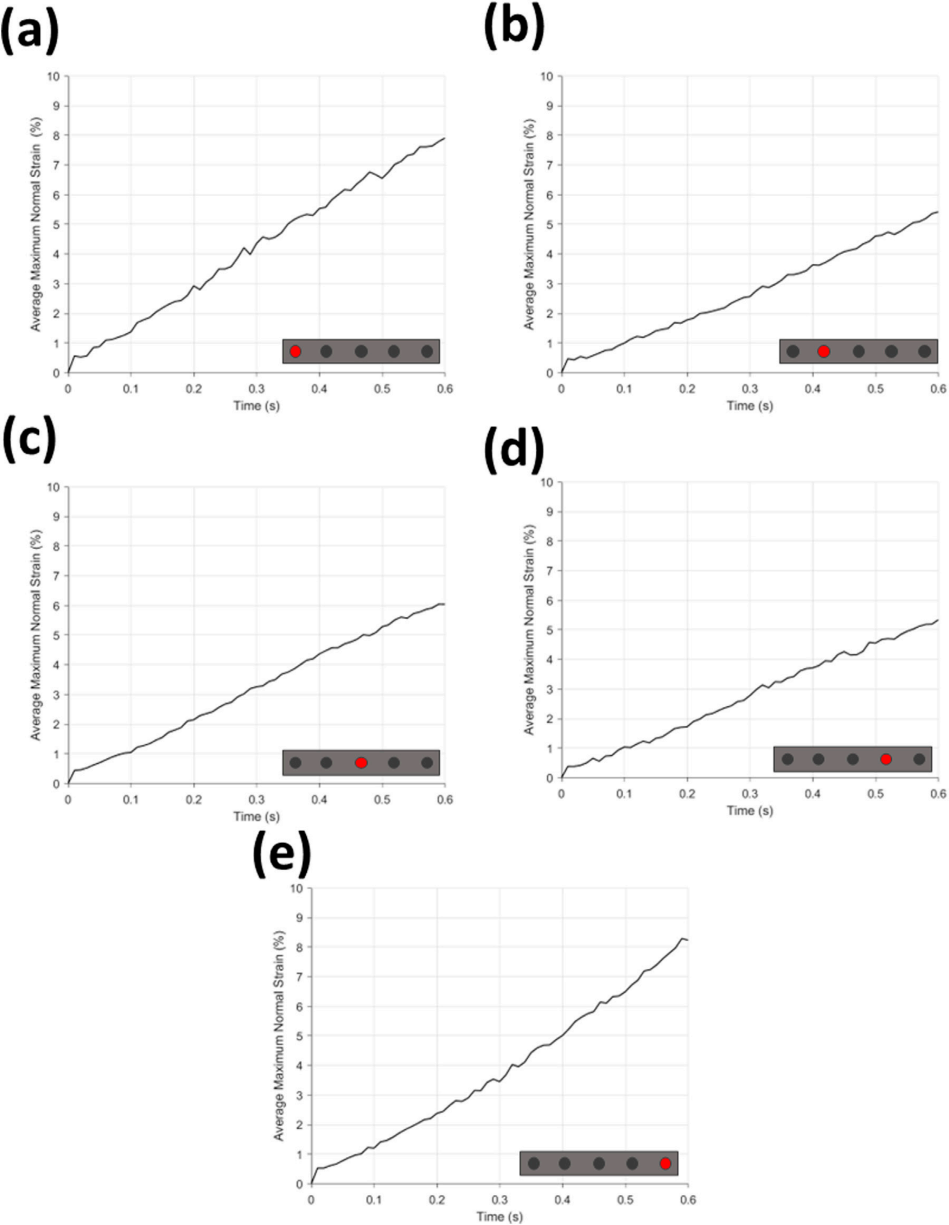
Average maximum normal strain (%) for **(a–e)** each microneedle position. Microneedle position can be found on schematic insert on graphs **(a–e)**.

Figure 8 presents a direct comparison of the maximum normal strain experienced at rupture (Figures 8a, c) and 100% (600 μm) and 30% insertion depth (Figures 8b, 9d) for MN and hypodermic needles respectively, with the same colour scale applied to both sample types. A slight difference in normal strain at rupture is visible, with MNs appearing to generate greater strain over their device area. Figure 8d shows the strain to increase in a localised area for the hypodermic needle sample, with multiple strain hotspots, peaking at an excess of 65%, whereas the MN sample (Figure 8b) peaks at full insertion well below this (12.86%).

**FIGURE 10:**
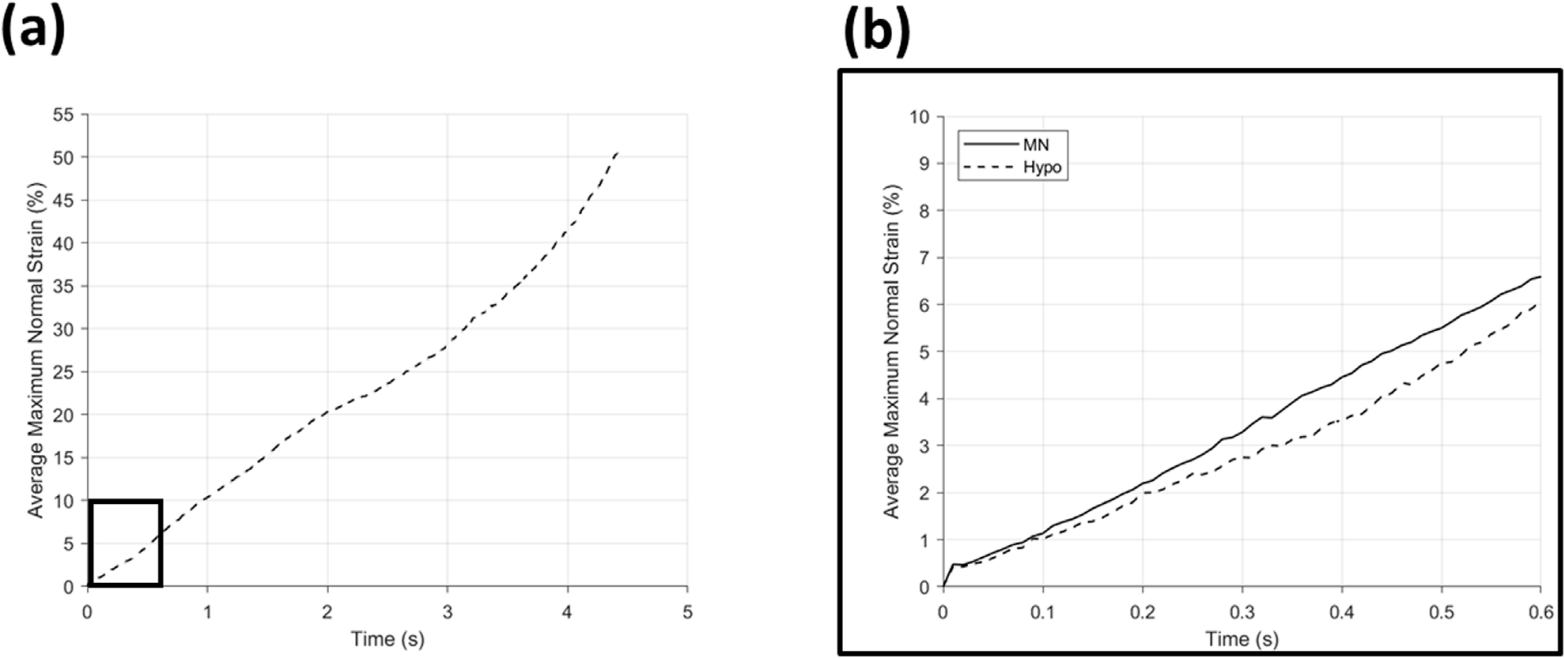
Average maximum normal strain (%) for **(a)** hypodermic needle insertion with a boxed area expanded into **(b)** showing a comparison of hypodermic needle (--) and microneedle (−) insertion over the entirety of the microneedle insertion time.

Found in Figure 9 are the graphs of average maximum normal strain during time for MN insertion grouped by needle position in array, and Figure 10a the same for hypodermic needle insertion. All graphs can be seen to have a continuous increase in average maximum normal strain across insertion time (0–0.6 s), and notably all demonstrate a sharp and repeatable peak between 0–0.01 s with a maximum and minimum strain value of 0.57% and 0.38% for the MN samples, and 0.42% for the hypodermic needle samples.

The average maximum normal strain relative to MN position can be seen in Figure 9; Table 5. The average of position 1 needles in MN arrays (Figure 9a) demonstrate a continuous incline from 0%–7.91% average maximum normal strain, with a notable degree of noise, specifically between 0.2–0.5 s. The average of position 5 needles in the MN arrays (Figure 10b) again depict a similar, continuous incline from 0%–8.24% at 0.6 s, with a similar degree of noise between 0.2–0.5 s. Notably, the difference in the average maximum normal strain values for position 1 and 5 needles is a minimal 0.33%.

**TABLE 5:** Maximum average maximum normal strain (%) for each microneedle position and hypodermic needle.

	Microneedle position					Hypodermic needle
	1st	2nd	3rd	4th	5th	
Maximum Average Maximum Normal Strain (%)	7.91	5.42	6.04	5.34	8.24	50.89

The average maximum normal strain of position 2 MN (Figure 9c) again demonstrates a continuous, although much more gradual incline in average maximum normal strain than needles in position 1 and 5, reaching a peak of 5.42%. The average maximum strain for position 2 MN (Figure 9c) also exhibit notably less noise than that demonstrated by positions 1 and 5 (Figures 9a, b). The average of MN in position 4 again demonstrate the same initial peak in strain between 0–0.01 s and reach an end peak of 5.34% maximum normal strain at 0.6 s, only 0.8% less than the peak of position 2. The degree of noise seen in Figures 9c, d appear to be analogous.

Figure 9e shows the average maximum normal strain of MN in position 3 of the array shares the same continuous increase in strain as the other four positions, peaking at 6.04% at 0.6 s, with minimal noise, comparable with position 2 and 4 (Figures 9c, d).

The average maximum normal strain of the hypodermic needle devices is plotted in Figure 10a, and despite the overall continuous increase in strain throughout the insertion, multiple phases can be identified: 0–2 s (0%–20.34%), 2–3.5 s (20.34%–34.14%), and 3.5–4.43 s (34.14%–50.89%) (Table 5). A comparison of the average maximum normal strain induced by MN and hypodermic needle insertion, over the MN insertion period, can be seen in Figure 10b, with an initial similarity in strain increase between 0–0.1 s, followed by a divergence until 0.5 s. Approaching the end of the full MN insertion at 0.6 s, the strain exerted by the two device types begins to diverge, before the hypodermic needle continues climb for the remainder of its full insertion time (0.6–5 s) to a peak of 50.89% (Figure 10; Table 5).

## Discussion

### Global mechanical response of MN versus hypodermic needles

Force-displacement studies are crucial with respect to needle-based devices, with it generally accepted that a reduced penetration force reduces pain experienced by patients (Leonardi et al., 2019). With respect to force-displacement curves, a consistent trend was displayed by MN devices between 0–0.22 mm (36% insertion depth), followed by a smooth gradual increase in force with no notable noise recorded (Figure 5a). This is crucial, as reliable and repeatable skin rupture and penetration between injections is paramount for patient compliance.

In comparison to the equivalent force-displacement curve for hypodermic needle devices, all repeats display the same oscillations in force between 0–0.2 mm (Figure 5b). The repeatable nature of these oscillations suggest this cannot be attributed to noise, and are hypothesised to be related to the singular, narrow point of contact for the hypodermic needle samples, combined with both local interactions such as sliding between the needle and membrane/PDMS interface, and resultant slippage from progressive loading. It does, however, reflect the repeatability of membrane rupture facilitated by hypodermic needles. A repeatable membrane rupture, designed to mimic the rupture of skin during needle insertion, is to be expected of a medical device which has regulatory approval and manufacturing quality control of the key insertion components including the bevel angle and lancet point (Ch et al., 2014).

With respect to MN insertion, it is important to recognise the role of compression in the consistent increase in force recorded following 0.4 mm displacement. The compression can be attributed to the array design, whereby five 600 μm MNs are connected by a flat silicon base plate, visible in the background of Figure 1b, which may continue to uniformly compress the insertion area following complete insertion of the MN shafts.

Comparatively, post membrane rupture and following 0.6 mm displacement (MN 100% insertion), little noise is observed for the hypodermic needle samples (Figure 5a), however greater variation is then seen between hypodermic needle samples when a larger proportion of the needle is travelling through the PDMS support plug, suggesting sliding plays a key role in insertions and is highly variable between insertions. Within a clinical setting, patients will have varying hydration levels, skin composition, and layer thickness within their skin barrier network, providing differing degrees of sliding to the needle path during insertion. Therefore, this experimental setup provides a fair representation of this variation. It has been reported that discomfort and pain is associated with the sliding force and local interactions that are associated with insertion which, as evidenced here, forms a greater component of hypodermic needle insertions due to the length of needle required to pass through the skin to achieve drug delivery depth (Leonardi et al., 2019).

### Digital image correlation

#### Comparison of strain progression and localisation during insertion

The strain fields generated in this study (Figures 6–8) provide a plethora of information to facilitate a better understanding of insertion profiles, and the continuous and evolving effect injections have on the skin. The strain fields for MN samples (Figure 6), demonstrate good repeatability, with a consistent range with respect to maximum normal strain at 100% insertion (9.88%–14.89%, stdev = 2.22). The standard deviation calculated at each stage of MN insertion are relatively consistent, with 2.63, 2.67, 2.22 for rupture, 50%, and 100% respectively. Furthermore, the resolution of the DIC processing depicts the strain discretised to specific needle points, contributing towards disproving the “bed of nails” theory for this sized array through variations in strain induced across the needle points, and not one rectangle of high strain spanning the array. Importantly, no facet loss was observed in the area of interest for any of the MN repeats, confirming the DIC conditions such as lighting and speckle quality, paired with the minimal deformation caused by the MN devices, allowed for good particle tracking. This novel method has afforded good resolution for the identification of each MN in the 1 × 5 array, and the variation in strain induced by each, further enabling the identification of the consistency and repeatability of insertion.

Conversely, the compressive elements of MN insertion due to the array structure contribute to noise around the devices particularly in repeat 1, making it difficult to decipher some needles from noise, with notably more background strain seen in the MN samples in comparison to the hypodermic needle (Figure 6). The greater degree of background strain may be partially due to local interactions and sliding between the membrane and the PDMS surface, in addition to the compressive design of the array structure. Furthermore, the greater background strain in the MN samples may be accounted for due to the resolution limitations and the small values of maximum normal strain registered during MN insertions lying closer to the background noise levels.

The hypodermic needle strain fields resulted in a highly localised maximum normalised strain in an area greater than just the lancet point (Figures 7, 8). Notably, minimal facet loss was observed considering penetration and a high degree of membrane tearing around the insertion point. Therefore, from a methodology perspective, good consistency with a good retention of facets was demonstrated throughout the testing, with only 1 facet loss in an area of interest, with a central facet in hypodermic needle repeat 3 lost at 29% insertion, just 1% earlier than the 30% required (Figure 7). Conversely to the MN samples, less background compression has been measured during hypodermic needle insertions, with sliding potentially contributing to the strain from membrane drag that cannot be discretised here from maximum normal strain, which is a flaw within this experimental design (Figure 8). Comparatively to the MN repeats, there is a marked increase in standard deviation variation within the pool of hypodermic needle repeats, with a standard deviation of 13.97 for 30% insertion depth. This could be accounted for by a variety of factors, including the greater insertion depth leading to the accumulation of greater variation within the method. It does, however, suggest that patients may experience a wide variety of strains, and therefore levels of pain throughout hypodermic needle injections. This unknown and varying pain levels may contribute to lack of patient compliance through fostering greater anxiety surrounding pre-injection expectations.

#### Rate of strain increase

Investigating and quantifying the rate of strain increase is crucial when studying needle insertion as it is important to understand how the strain changes over the duration of administration. This will allow an insight into how effects such as local effects including sliding, skin/membrane relaxation, penetration depth, collectively contribute to the rate of strain increase. Rate of strain increase provides an insight into user experience, with more significant and steeper increases commonly associated to greater degrees of pain and discomfort during insertion, and resultantly poorer patient experience. Beneficially, the method developed and reported in this paper allows the isolation of the area around each needle tip, enabling investigation into how the direct area around the rupture point is affected with respect to insertion time. With respect to device development, this enables the identification of any discrepancies or trends with respect to insertion and needle position which can feed back into modifications and inform future design choices.

When using maximum normal strain as a performance indicator, it becomes clear that there is a trend in the strain generated by each individual devices on the MN array. MNs which are position 1 and 5 produce a similar gradient and noise level with respect to rate of strain increase, peaking at similar values of 7.91% and 8.24% respectively (Figures 9a, b; Table 5). Furthermore, the average maximum normal strain generated by MNs in positions 2 and 4 also show a similar trend, peaking at 5.42% and 5.34% respectively (Figure 9c, d; Table 5). Lastly, with respect to devices in position 3, the array’s central needle, the average maximum normal strain value peaks at 6.04% (Figure 9e; Table 5). These results suggest pinning of the membrane is occurring between the needles on the extremities of the MN device, with the first and fifth needle is pinning the membrane, supported by the central needle. Pinning refers to the mechanism where the membrane is held or restrained between the microneedles, preventing further displacement or movement at these specific locations during the insertion process, identifiable by decreases in strain at certain needle positions. Resultantly, the skin phantom has been held away from needles in position 2 and 4, limiting their penetration potential, and therefore the effectiveness of the devices in those positions. With respect to influencing device design choices, this may suggest that pitch alterations are required to maximise the efficiency of device insertion.

With specific respect to the hypodermic needle samples, investigating the rate of strain increase allows for a better understanding of the impact of the length of device on strain around the injection site. For example, comparing both the average for the hypodermic needle and MN devices at 0.6 s into the insertion (0.6 mm insertion depth), the maximum normal strain is measured to be 6.07% and 6.59% respectively, suggesting that when compared at the same insertion depth, MN to induce 0.52% greater strain when compared to hypodermic needle-mediated injections (Figure 10; Table 5). It is important, however, to acknowledge that this comparison point represents a MNs full insertion depth, as opposed to 30% of the hypodermic needle’s length, which represents just 3.75% of the complete insertion depth. This finding, in conjunction with the initial force-displacement graphs, supports that the hypodermic needle devices have a sharper lancet point, and require less immediate force for penetration (Figures 5c, 9). However, considering the maximum normal strain achieved across the entirety of the test, it is apparent the hypodermic needles induce greater strain on account of its invasiveness, predominantly due to the device length and gauge (shaft width) (Figures 6–8).

### Limitations and future work

As with the development of any new methodology, certain limitations are to be expected, and these provide important insights and direction for future work. Despite capturing all key deformations, a potential limitation within this study is the lack of frames collected of the hypodermic needle’s full insertion depth. The practical reason for this flaw was due to constraints surrounding data capture and storage, with the number of frames required to capture full 16 mm insertion proving very computationally expensive. Future work could see full insertion captured; however, this would require a trade-off of either a reduced frame rate compared to this work, or a variable frame rate, with a higher frame rate used for rupture and lower for insertion succeeding this.

Additionally, the homogeneity of the membrane surface and the lack of topical skin features, such as hair and pores, limit the mimicking capabilities of the skin phantom to elasticity and stratum corneum-akin thickness. To better mimic additional skin features, *ex vivo* porcine skin could be used in future work, however for the most robust investigation, *ex vivo* human skin should be used.

Future work investigating various MN composition, geometries, and pitch is suggested to examine and quantify the effects these may resultantly have on the strain, with specific interest into how alterations in pitch may reduce the membrane pinning and improve insertion efficiency. This test method provides a valuable tool for evaluating microneedle (MN) designs, offering insights that can guide the optimization of their geometry and array structure. By using this approach, future iterations can be refined to minimize tissue disruption while maximizing efficiency in needle insertion. This could ultimately lead to more effective and minimally invasive MN designs. Lastly, to validate this method, an interesting piece of future work could pair this study with a visual analogue scale mediated set of repeats on patients/volunteers in an attempt to validate the link between strain and pain.

## Conclusion

A novel method has been reported here using stereo-DIC to quantify the strain experienced by a skin phantom following hypodermic needle and MN mediated insertions. This methodology has allowed objective quantification of strain, unbiased by individual interpretation and other flaws generally associated with the most commonly employed methodology used in these studies, VAS. Force-displacement investigations suggested hypodermic needles to be sharper than MNs, however MN devices have displayed that they unequivocally exert less strain than hypodermic needles over the maximum insertion depth measured in this study. Lastly, with respect to device performance, isolating each MN point with respect to change of strain over time has highlighted key design modifications for future versions of the MN arrays, namely, a greater needle pitch. This test method can be used to assess MN design and inform future iterations, aiding in the redesign of potentially a more optimal geometry and array structure for minimally disruptive tissue loading. Improvements could be made to this methodology to increase robustness and improve skin mimicry; however, this methodology provides a good platform to be employed for the objective and non-invasive quantification of strain following needle insertions.

## Data Availability

The raw data supporting the conclusions of this article will be made available by the authors, without undue reservation.
